# Current management of tubal infertility: from hysterosalpingography to ultrasonography and surgery

**Published:** 2015

**Authors:** I Briceag, A Costache, VL Purcarea, R Cergan, M Dumitru, I Briceag, M Sajin, AT Ispas

**Affiliations:** * Department of Obstetrics and Gynecology, “Cantacuzino” Clinical Hospital, Bucharest, Romania; ** Ultrasound Teaching Center, “Carol Davila” University of Medicine and Pharmacy, Bucharest, Romania; *** Marketing and Medical Technology Department, “Carol Davila” University of Medicine and Pharmacy, Bucharest, Romania; **** Anatomy Department, “Carol Davila” University of Medicine and Pharmacy, Bucharest, Romania; ***** ”Carol Davila” University of Medicine and Pharmacy, Bucharest, Romania; ****** Pathology Department, “Carol Davila” University of Medicine and Pharmacy, Bucharest, Romania

**Keywords:** tubes, infertility, hysterosalpingography, ultrasonography, laparoscopy

## Abstract

**Rationale.** The development of IVF techniques has diminished the importance of tubal infertility but recent discoveries shed a new light on reproductive tubal surgery prior to any IVF cycle.

**Objective.** To adapt current state of the art recommendations concerning tubal factor infertility to actual possibilities in Romanian healthcare system and to grow the awareness of fellow fertility specialists and general practitioners to the improved outcomes of novel management and treatment modalities.

**Methods and results.** 67 free full text articles centered on the subject of management in tubal infertility were identified in international databases. Four articles described general diagnosis using data from medical history, 21 works approached the diagnosis through hysterosalpingography, 14 papers introduced the use of different sonographic procedures, 8 files analyzed the importance of exploratory laparoscopy and 20 articles reviewed different treatment modalities.

**Discussions.** Current data show that active implementation of the large scale use of tubal surgery prior to any IVF cycle will reduce up to 30% the costs associated with obtaining a viable pregnancy in cases with tubal factor sterility.

## Introduction

The problem of fertility is growing nowadays not only in western countries but also in other areas as even in developing countries the couples postpone childbirth well over the age of 30 in order to focus on their careers and financial stability [**1**]. As seen in many guidelines the gold standard end point of care when speaking about infertility are the IVF techniques, but these are still expensive and present low accessibility in many countries and needless to mention that in some instances there is the barrier of culture and religion that needs to be broken [**2**]. Main causes of tubal factor infertility are: tubal obstruction or occlusion (proximal, distal, unilateral or bilateral) [**3**], endosalpingeal destruction [**4**], periadnexal adhesions [**5**], pelvic inflammatory disease [**6**], endometriosis [**7**], ectopic pregnancy [**8**], abdomino-pelvic surgery, use of intrauterine devices, induced surgical abortion [**9**], etc.

## Methods

International databases were queried for articles on the subject of management in tubal infertility. Therefore, 67 free full text articles were found on this subject with the following distribution concerning the main subjects: 4 articles on general diagnosis using data from medical history, 21 works concerning hysterosalpingography, 14 papers on the use of different sonographic procedures, 8 files on the importance of exploratory laparoscopy and 20 articles analyzing different treatment modalities. Given the fact that up to 30% of the cases with infertility have a tubal factor we hope to adapt current practice to actual resources in Romania in order to expedite the diagnosis and reduce costs with treatment and better manage the already low accessibility to IVF techniques.

## Results

In case of suspected tubal infertility women who are not known to have comorbidities (PID, history of ectopic pregnancy or endometriosis) should be offered hysterosalpingography as initial screening test; alternatively hysterosalpingography should be replaced with hysterosalpingo-contrast-ultrasonography if available and in case of associated comorbidities the patient should be subjected to laparoscopic chromopertubation [**10**].

Hysterosalpingography is the fluoroscopic visualization of the uterine cavity and fallopian tubes by injection of a radio-opaque contrast media and is credited with 84% sensitivity and 74.5% specificity [**11**]. Tubal spasm is the culprit for lower accuracy of this diagnostic imaging technique but with the use of intravenous scopalamin and rotation of the patient it has been reduced to a minimum [**12**]. HSG has two contraindications: PID and pregnancy (always perform the procedure during days 7-12 of the cycle), and a history of allergic conditions requires premedication with methylprednisolone 32mg for 12h and 2h in advance [**13**]. Moreover HSG with an oil-based contrast media has been proven to have a somewhat therapeutic role through flushing of tubal debris [**14**].

Sonohysterography and hysterosalpingo-contrast sonography are credited with 84.6% sensitivity and 99.7% specificity in detecting hydrosalpinx [**15**]. Hysterosalpingo-contrast sonography evolved from using a negative contrast which is saline water to a positive contrast agent that is microbubble agent and from 2D to 3D and even 4D imaging [**16**]. HyCoSy is advantageous as patients exhibit a better pain tolerance, avoidance of iodinated contrast medium and preventing the use of ionizing radiation [**17**].

**Fig. 1:**
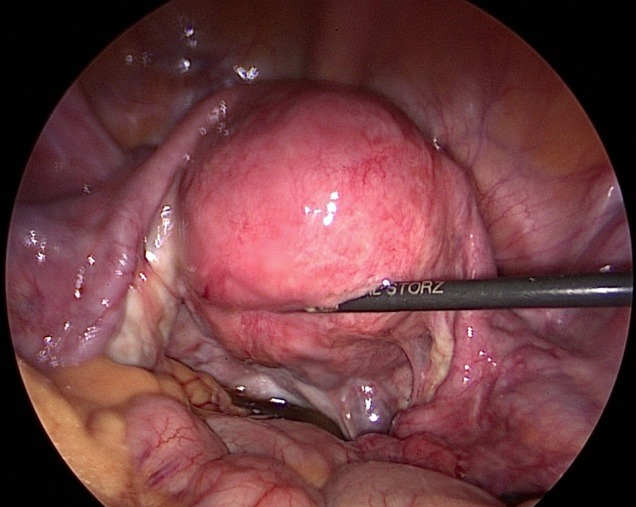
Normal laparoscopic aspect of the female reproductive organs

**Fig. 2:**
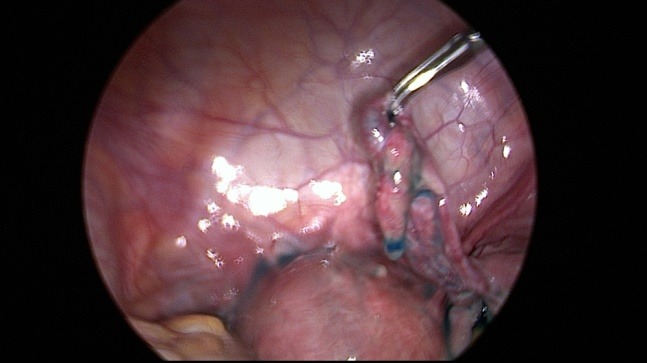
Tubal patency test with blue dye

**Fig. 3:**
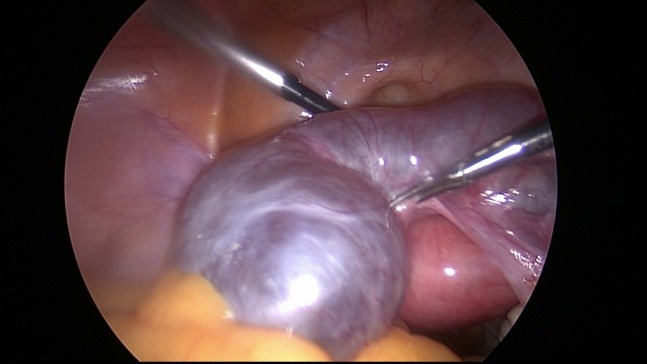
Laparoscopic view of bilateral hidrosalpinx

Unfortunately laparoscopic chromopertubation remains the gold standard in evaluating the tubal sterility (**Fig. 1**) [**18**]. Through the injection of diluted indigo carmine into the uterine cavity with simultaneous laparoscopy in order to visualize the tubal fill and spill into the abdomen (**Fig. 2**) [**19**]. The disadvantages of this procedure are: expensive, invasive and requires anesthesia (**Fig. 3**) [**20**].

## Discussions

For proximal tubal disease other macroscopic anastomosis surgeries have been abandoned in favor of selective salingography and transcervical tubal cannulation. Although observational studies have credited this procedure with up to 85% success, there are still no RCTs compared with no active treatment, and the rate of ectopic pregnancy was of 9% and the risk of tubal perforation of 2% [**21**].

In cases with distal tubal disease is offered the option of a neosalpingostomy which can be technically upgraded till the level of laparoscopy with CO2 or yttrium aluminum garnet laser used for performing a cruciate incision at the distal end of the tube [**22**].

There are still some unclear aspects concerning the association between hydrosalpinx and tubal sterility but the introduction of salpingectomy prior to IVF procedures has improved overall outcome of IVF procedures and thus proved 2.4 fold increase in the delivery rate [**23**].

Any diagnostic laparoscopy should be immediately followed in the same session by the lysis of adhesions and that this simple procedure is followed by a 60% conception rate in the following 6 to 12 months [**24**].

A distinct group of patients with tubal infertility is that requiring sterilization reversal through tubal reanastomosis. It has been proven that the outcome of such a procedure is foreseen by the initial sterilization method used thus laparoscopically sterilized patients had 50% chances of conception as laparoscopic sterilization requires minimal injury to the tube [**25**].

## Conclusions

Reproductive surgery has the objective of restoring the natural procreation in couples with tubal factor infertility. At worldwide level, there is still need for a randomized control trial comparing the benefits and costs for IVF versus reproductive tubal surgery. Unfortunately, in Romania the expertise chain needed for a complete fertility clinic is broken at the level of diagnosis through sonography as in the evidence of the Romanian Society for Ultrasonography in Medicine and Biology there are registered only 3 healthcare specialists able to perform hysterosalpingo-contrast sonography. Given the fact that nowadays a single IVF cycle costs around 2600 U.S. $ and the estimated cost of a reproductive tubal surgery prior to IVF is of 1000 U.S. $ and that it was established that reproductive tubal surgery can half the number of IVF cycles needed, one can simply imagine a minimum of 30% cost reduction by associating reproductive tubal surgery with a follow-up of 6 months prior to first IVF cycle. 

**Disclosures**

None
